# Effects of Heating and Cooling of Injection Mold Cavity Surface and Melt Flow Control on Properties of Carbon Fiber Reinforced Semi-Aromatic Polyamide Molded Products

**DOI:** 10.3390/polym13040587

**Published:** 2021-02-15

**Authors:** Yasuhiko Murata, Ryota Kanno

**Affiliations:** 1 Department of Mechanical Engineering, Faculty of Fundamental Engineering, Nippon Institute of Technology, 4-1 Gakuendai, Miyashiro-machi, Minamisaitama-gun, Saitama 330-8501, Japan; 2 Mechanical Systems Engineering Major, Graduate School Nippon Institute of Technology, 4-1 Gakuendai, Miyashiro-machi, Minamisaitama-gun, Saitama 330-8501, Japan; 2197011@stu.nit.ac.jp

**Keywords:** heating and cooling of injection mold, melt flow control, carbon fiber reinforced semi-aromatic polyamide, fiber orientation, bending strength, weld line, crystallization

## Abstract

Fiber reinforced thermoplastics (FRTP), reinforced with glass or carbon fibers, are used to improve the mechanical strength of injection-molded products. However, FRTP has problems such as the formation of weld lines, the deterioration of appearance due to the exposure of fibers on the molded product surface, and the deterioration of the strength of molded products due to the fiber orientation in the molded products. We have designed and fabricated an injection mold capable of melt flow control and induction heating and cooling. This mold can both heat and cool the injection mold. It can also control the melt flow direction using a movable core pin. In this study, the above-mentioned mold was used for the molding of carbon fiber reinforced semi-aromatic polyamide. As a result, we found that increasing the heating temperature of the mold and increasing melt flow control volume contribute to the prevention of the generation of a weld line and the exposure of fibers on the molded product surface, as well as to the formation of a flat surface and increased bending strength. The relationships of these results with the carbon fiber orientation in the molded products and the crystallization of semi-aromatic polyamide were also examined in this study.

## 1. Introduction

**Figure 1:**
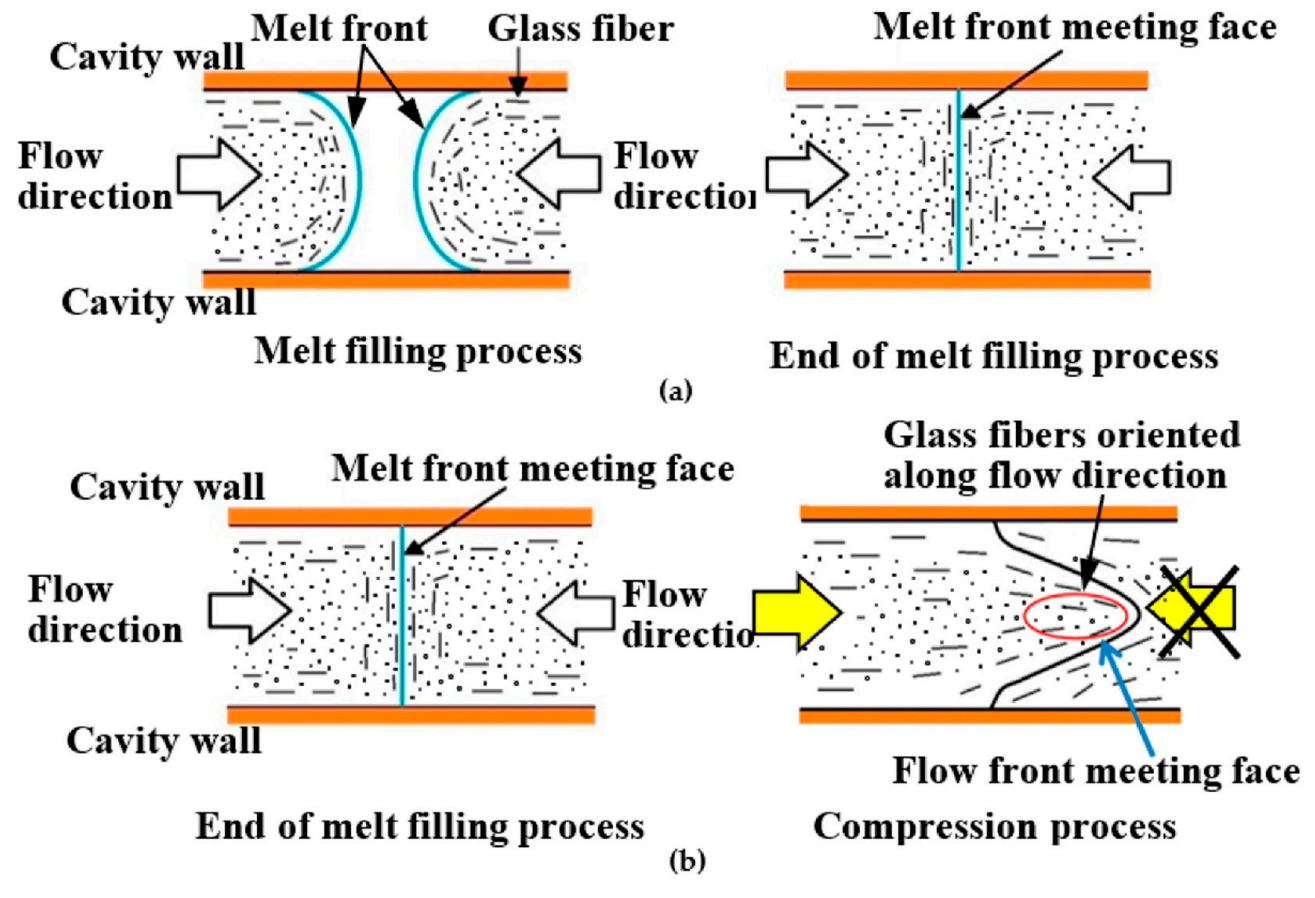
Fiber orientation around melt front meeting area: (**a**) schematic diagram of the melt front meeting area; (**b**) method of improving strength in the melt front meeting area.

Polymers are light with excellent shapability. However, they have lower mechanical strength than metals. To address this problem, fiber reinforced plastics (FRP), which are reinforced with glass or carbon fibers, have been put to practical use and applied to structural members where high mechanical strength is required. Recently, the injection molding of fiber reinforced thermoplastics (FRTP) containing short fibers has been widely carried out to mass-produce molded products with complex shapes. In injection molding, a V-notch-shaped weld line [1] is generated in the melt front meeting area. This is where the melt fronts divided by an insert, such as a pin or a block, in a mold cavity rejoin each other downstream of the insert. The weld line degrades the appearance of molded products. As shown in Figure 1a, fibers in an FRTP are locally oriented parallel to the melt front meeting area, i.e., along the thickness direction of molded products, leading to the occurrence of anisotropy. The local deterioration of strength in the melt front meeting face results in the reduced strength of the molded products [2,3]. In addition to the weld line, the exposure of fibers on the molded product surface results in a rough surface, which degrades the appearance of the product. Various measures are taken at production sites to address these problems. Rapid heat and cool injection molding is an example of a measure to prevent the generation of weld lines. In a mold, a cooled and solidified thin layer, called a skin layer, is formed at the moment the melt at the melt front forms a fountain flow and comes in contact with the cavity wall, which is kept at a low temperature. It has been clarified that a weld line is generated on the surface of a molded product, and the transcription of the cavity wall onto the surface of a molded product fails while the melt at the melt front meeting area forms a skin layer [4]. Therefore, the formation of a skin layer should be delayed; concretely, the cavity wall is heated to the glass transition point T*g* or higher to ensure that the melt flowing near the cavity wall does not transform from rubber state to glass state [5]. When the melt is pressed to the cavity wall in this state upon the application of melt pressure and then cooled, the V-shaped groove of the weld line disappears and the transferability is improved, as in a previous report [6]. Variations of this method include rapid heating cycle molding (RHCM) [7], which circulates hot water or water vapor within the mold; the electric heater method, which heats the mold using a cartridge, sheath, or ceramic heater embedded in the mold [8,9]; and the induction heating method, which uses electromagnetic induction [6,10,11]. On the other hand, the local deterioration of strength in the melt front meeting area is prevented by the method shown in Figure 1b. With this method, one of the two flows is blocked immediately after the two melt fronts meet and the melt is allowed to flow only from the other direction. This induces an internal melt flow that goes through the meeting face, causing the fibers near the meeting face to orient parallel to the melt flow direction. As a result, the deterioration of strength is reduced because the fibers increase resilience against tensile and bending forces. The suggested methods to induce such an internal melt flow are as follows: (1) the push–pull method [12], with which the melt is injected alternately from two injection cylinders, (2) shear-controlled orientation in injection molding (SCOLIM) [13], which uses a passage-switching device placed between the injection cylinder and the mold, (3) the press α method [14], which drives a core block or a core pin into the mold, (4) the rotary core method [15], (5) the in-mold core pin driving method [16], and (6) the rotary runner exchanger method [17].

However, there have been few detailed reports on how to achieve all the above purposes simultaneously, namely, the prevention of the generation of a weld line in the melt front meeting area and the exposure of glass fibers on the molded product surface as well as the prevention of the deterioration of strength due to the local orientation of fibers, although an attempt has been made to improve both the appearance and strength of molded products by combining the heating of the mold by RHCM with core driving [18]. The effects of the heating conditions of the mold and the conditions of core driving on the strength and appearance of molded products and the orientation of glass fibers have not been systematically evaluated.

We newly designed and fabricated an injection mold capable of both melt flow control using a movable core pin and induction heating and cooling at the same time. Using this mold, we evaluated the effects of differences in the heating conditions of the mold and the conditions of melt flow control on the appearance, fiber orientation, and mechanical strength of molded products made of polypropylene reinforced with short or long glass fibers [19].

In this study, the above-mentioned mold was applied to the molding of short carbon fiber reinforced semi-aromatic polyamide which has already been widely used for automotive components because of its excellent mechanical strength, heat resistance, and dimensional stability. Then, the effects of the heating and cooling conditions of the mold and the conditions of melt flow control on the appearance and mechanical strength of molded products were evaluated.

## 2. Experiment

### 2.1. Molding Process and Mold Structure

**Figure 2:**
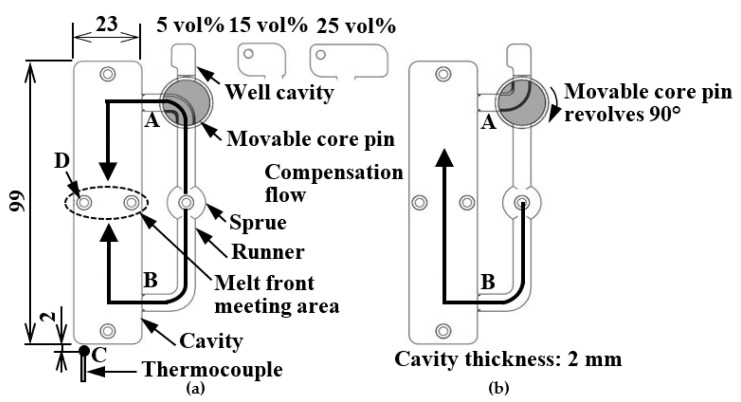
Method of melt flow control (unit: mm): (**a**) melt filling process; (**b**) holding pressure process.

Figure 2 shows the method of controlling the melt flow in the melt front meeting area (hereinafter referred to as “melt flow control”). The rotary runner exchanger method [17] was adopted in this mold. The long rectangular cavity used in this study had dimensions of 99 (L) × 23 (W) × 2 (T) mm and two-point gates facing each other. A movable core pin, which was rotationally driven, was placed in the middle of a runner to switch the direction of the flow channel from the cavity to the well cavity. First, the cavity surface was induction-heated to a certain temperature and then the melt was injected into the cavity. Measures were taken to prevent the generation of a weld line in the melt front meeting area and to improve the surface property of molded products in the melt filling process shown in Figure 2a. After the melt fronts met, the induction heating was stopped and cooling water at a certain temperature was introduced into the mold in the holding pressure process shown in Figure 2b. Then, the melt flow from Gate A was blocked and the flow channel was directed to the well cavity by rotating the movable core pin by 90°. The melt flow was further directed to the well cavity by the compensation flow from Gate B, which was generated by the holding pressure. As a result, an internal melt flow occurred in the melt front meeting area. The aim of inducing such an internal melt flow was to improve the appearance and strength of molded products through the control of the fiber orientation in the products. As a reference for the comparison of strength, molded products obtained by one-point gate molding were fabricated by fixing the movable core pin in the position shown in Figure 2b throughout the injection molding process.

**Figure 3:**
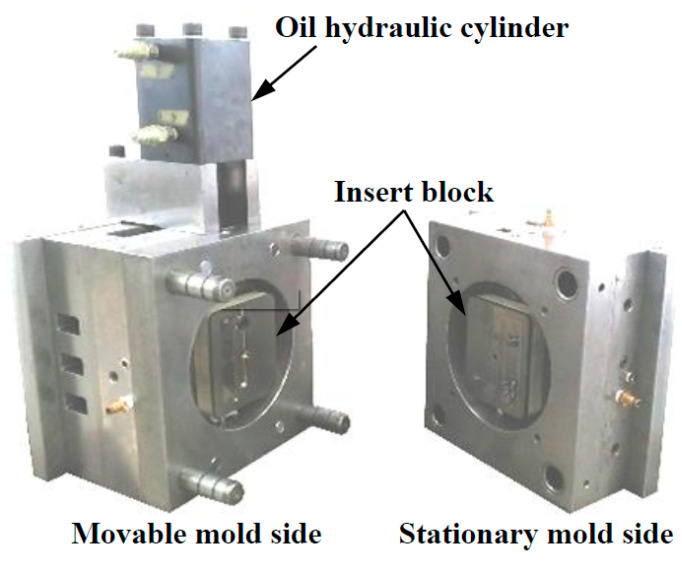
Appearance of the injection mold capable of melt flow control and induction heating and cooling.

**Figure 4:**
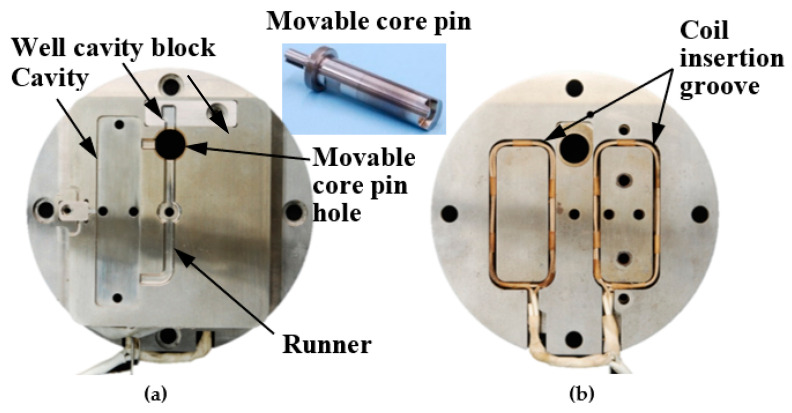
Appearance of the cavity insert and movable core pin: (**a**) front view; (**b**) back view.

Figure 3 shows the appearance of the injection mold capable of melt flow control and induction heating and cooling. An electromagnetic induction coil was embedded in each of the insert blocks placed on the stationary and movable mold sides. A cavity insert equipped with the movable core pin and an oil hydraulic cylinder were installed on the movable mold side. The movable core pin was rotated using this oil hydraulic cylinder to switch the direction of the flow channel. Figure 4 shows the appearance of the cavity insert and movable core pin. The movable pin was inserted in the hole located midway along the runner. The induction coils were installed in the rectangular grooves at the back of the insert.

The temperature of the cavity insert was controlled on the basis of the temperature measured using an alumel–chromel sheath thermocouple (⌀ 1.6 mm), which was inserted at point C in Figure 2. The volume of the well cavity used in this study was 5, 15, or 25 vol% of the volume of the mold cavity as shown in Figure 2. The effects of these three melt flow control volumes on the appearance, fiber orientation, and strength of molded products were examined while changing the well cavity.

### 2.2. Experimental Method

A stationary induction heater (SK-NF002SA; Ju-OH Inc., Hiratsuka, Japan) was used for the induction heating of the mold. A mold temperature controller (TYPE TA-32; Stolz Co., Ltd., Murayama, Japan) was used for cooling the mold. An injection molding machine (ROBOSHOT S-2000 i50A; Fanuc Ltd., Oshino, Japan) with a maximum clamping force of 500 kN was used in the experiments. The polymer used in the experiments was short carbon fiber reinforced semi-aromatic polyamide MXD6 (Reny C36-B43; carbon fiber content, 30 wt%; Mitsubishi Engineering-Plastics Corporation, Tokyo, Japan).

Table 1 shows the molding conditions. Normal molding was carried out by maintaining the insert temperature at 95 °C. Heat and cool molding was carried out by injecting the melt into the cavity after increasing the insert temperature from 95 to 140, 160, and 180 °C, stopping the heating after the injection, and decreasing the insert temperature to 95 °C. Because the T*m* of the semi-aromatic polyamide used in this study was 243 °C, the solidification of melt in the cavity was not completely inhibited under the above heating conditions during the molding process. The rotation of the movable core pin was started at the start of the holding pressure process. The start time of melt flow control was set as the start time of the rotation of the movable core pin. Melt flow control was started manually by an operator who was checking the waveform of melt pressure obtained using an indirect quartz pressure transducer (Type 9221; Kistler Japan Co., Ltd., Yokohama, Japan) inserted immediately below ejector pin D in Figure 2. The start time of melt flow control varied by ±0.1 s because the melt flow control was started manually.

The effect of the difference in melt flow control volume on the strength of molded products was examined using three well cavities of different volumes as described above. To examine the effects of the differences in the cooling conditions of the mold on the strength of molded products, the cooling water temperature circulated within the mold was changed to 20, 50, and 80 °C. The cooling of the mold was started at the start of the holding pressure process.

**Figure 5:**
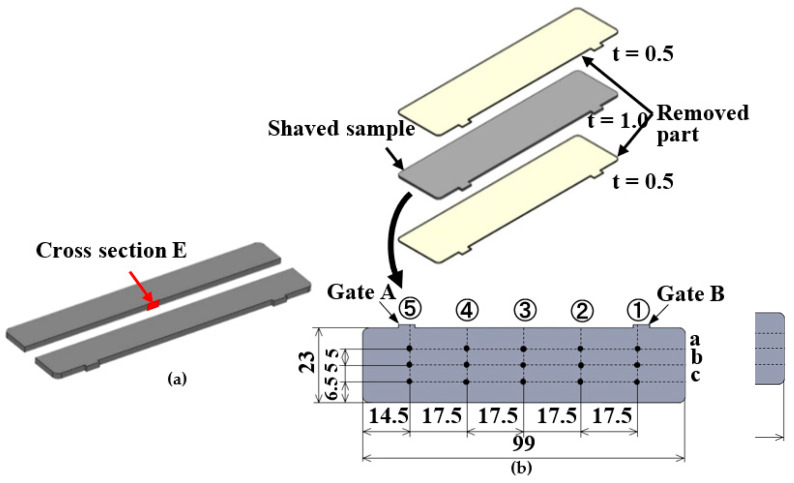
Observation positions of fiber orientation: (**a**) observation area along the thickness direction; (**b**) observation position on the shaved sample (unit: mm).

The appearance and cross sections of the molded products were observed and the surface shape was measured using a shape analysis laser microscope (VK-9700; Keyence Corporation, Osaka, Japan). The fiber orientation was observed on cross section E along the thickness direction of the molded products as shown in Figure 5a. The distribution of the fiber orientation in the molded products at arbitrary positions was quantitatively determined using a fiber orientation identification and evaluation system (Bethel Co., Ltd., Tsuchiura, Japan), which was based on the periodic heating and infrared radiation thermometer method [20]. With this method, the distribution of the fiber orientation was determined on the basis of the diffusion of heat applied periodically to a molded product. To exclude the effect of the fiber orientation in the skin layer formed near the molded product surface, the distribution of the fiber orientation was determined in samples of 1 mm thickness obtained by polishing and removing the top and bottom layers, each of 0.5 mm thickness, from the molded products as shown in Figure 5b. The distribution of the fiber orientation was observed at the points of intersection of lines ①–⑤ and lines a–c. The obtained results showed the average distribution of the fiber orientation along the thickness direction observed at the above points of intersection of the 1-mm-thick samples.

A universal tester (Tensilon RTC-1225A; Orientec Co., Ltd., Tokyo, Japan; maximum load cell force, 2.5 kN) was used to measure the bending strength of the molded products. A three-point bending test was performed by applying load through indenters pressed onto line ③ in the melt front meeting area as well as lines ② and ④ in Figure 5b to determine the bending strength at each point. The distance between the supporting points is 32 mm and the bending speed is 2 mm/min.

## 3. Results

### 3.1. Results of Observing Molded Product Surface

**Figure 6:**
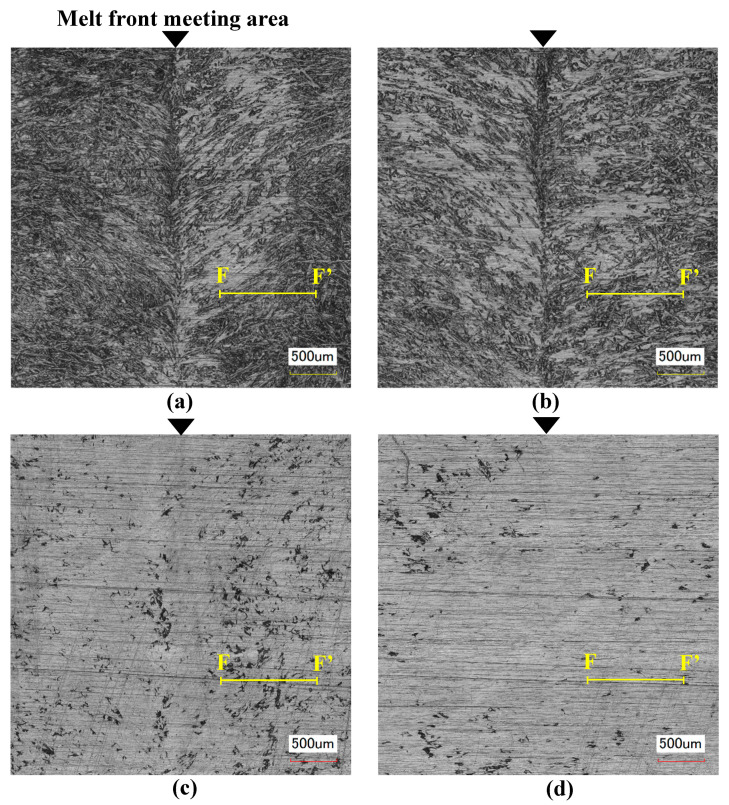
Appearance of surface of molded products: (**a**) without flow control (95 °C, surface roughness Ra; 3.466 μm); (**b**) with flow control: 0s (95 °C, melt flow control volume: 15 vol%, surface roughness Ra; 3.454 μm); (**c**) without flow control (95→180→95 °C, surface roughness Ra; 1.706 μm); (**d**) with flow control: 0s (95→180→95 °C, melt flow control volume: 15 vol%, surface roughness Ra; 1.420 μm).

**Figure 7:**
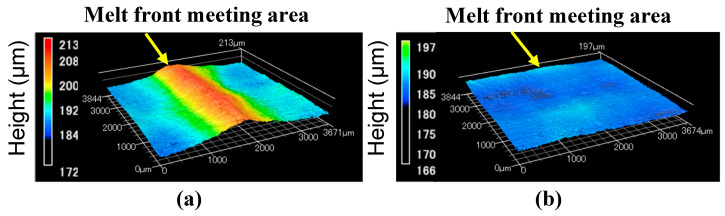
Surface shape around melt front meeting area of molded products (95→180→95 °C): (**a**) without flow control; (**b**) with flow control (melt flow control volume: 15 vol%).

Figure 6 shows the results of observing the surface in and around the melt front meeting area of the molded products. In Figure 6, the surface roughness Ra measured along line F to F’ is given. Figure 6a and b shows the surfaces of the molded products obtained by normal molding at a constant temperature of 95 °C. Fibers were exposed and a weld line was generated on both surfaces of the molded products obtained by two-point gate molding without (Figure 6a) and with (Figure 6b) melt flow control. A V-notch was not formed in these molded products, but the meeting face in the melt front meeting area where the fiber orientation changes appeared as a weld line. These results indicate that melt flow control does not affect the molded product surface when the mold is not heated. Figure 6c and d shows the surfaces of the molded products obtained by heat and cool molding (95 °C→180 °C→95 °C). The weld line disappeared and the exposure of fibers was reduced. The molded product surface was smooth regardless of whether or not melt flow control was performed. The surface of the molded product obtained with melt flow control (Figure 6d) was slightly smoother than that without melt flow control (Figure 6c). In particular, the surface of the molded product obtained with melt flow control (Figure 6d) showed the smallest Ra among (6a–d) and was smooth. The surface shape in the melt front meeting area of the molded products obtained by heat and cool molding (95 °C→180 °C→95 °C) was observed using a shape analysis laser microscope. The results are shown in Figure 7. An undulating shape with the melt front meeting area as its apex was observed on the surface of the mold product obtained by two-point gate molding without melt flow control (Figure 7a). On the other hand, the surface of the mold product obtained by two-point gate molding with melt flow control (Figure 7b) was flat and did not have an undulating shape. Similar results were also obtained when the injection molding was carried out without heating the mold, which revealed that heating the mold had no effect on the formation of the undulating shape.

As discussed above, the exposure of fibers and the generation of a weld line could be prevented by heating the mold. A flat surface without an undulating shape could be achieved by melt flow control regardless of whether or not the mold was heated.

### 3.2. Bending Strength

**Figure 8:**
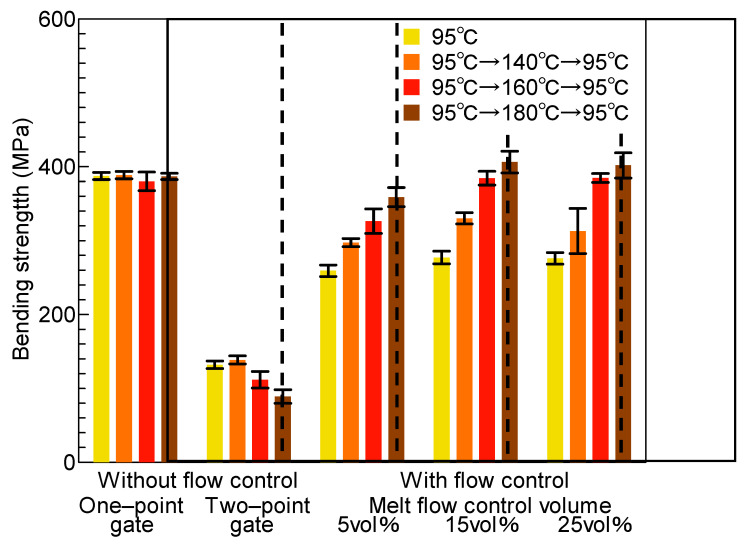
Bending stress of the molded products (indenter position ③).

A three-point bending test was performed by applying load through the indenter pressed onto line ③ in the melt front meeting area in Figure 5b. Figure 8 shows the bending strength properties of the molded products obtained under different molding conditions. The bending test was carried out for five molded products under each molding condition. The mean and standard deviation (1σ) of the bending strength for the five molded products were calculated and plotted on a graph. The bending strength of the molded products obtained by two-point gate molding without melt flow control slightly decreased as the heating temperature of the mold increased. This result indicated that, although the heating of the mold contributed to the disappearance of the weld line, it did not improve the bending strength in the melt front meeting area when melt flow control was not performed. The bending strength of the molded products obtained by two-point gate molding with melt flow control was nearly threefold that of the molded products obtained by two-point gate molding without melt flow control and was comparable to that of the molded products obtained by one-point gate molding. The bending strength also increased as the heating temperature of the mold increased when melt flow control was performed. Moreover, the bending strength increased as the melt flow control volume increased for all heating temperatures of the mold.

**Figure 9:**
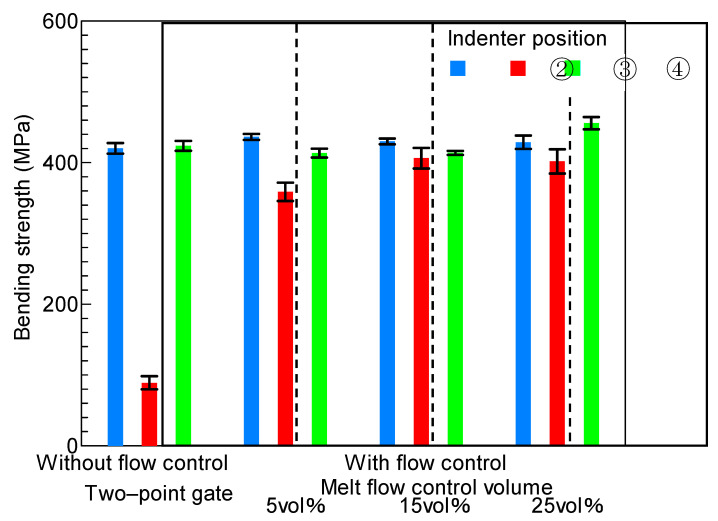
Bending stress of molded products obtained by changing indenter position (95→180→95 °C).

A three-point bending test was performed by applying load through the indenters pressed onto lines ②, ③, and ④ of the molded products obtained by heat and cool molding (95 °C→180 °C→ 95 °C). Figure 9 shows the bending strength measured by the three-point bending test. On line ③ in the melt front meeting area, the bending strength increased sharply as the melt flow control volume increased. The bending strength on line ③ was close to that on lines ② and ④ when the melt flow control volume was more than 15 vol%. On line ②, the bending strength did not change and remained high even when the melt flow control volume increased. The bending strength was high on line ④. The bending strength on line ④ of the molded products obtained by two-point gate molding and that of the molded products obtained with melt flow control volumes of 5 and 15 vol% were almost the same. The bending strength on line ④ slightly increased when the melt flow control volume was 25 vol%.

### 3.3. Cooling Conditions of Mold and Bending Strength

**Figure 10:**
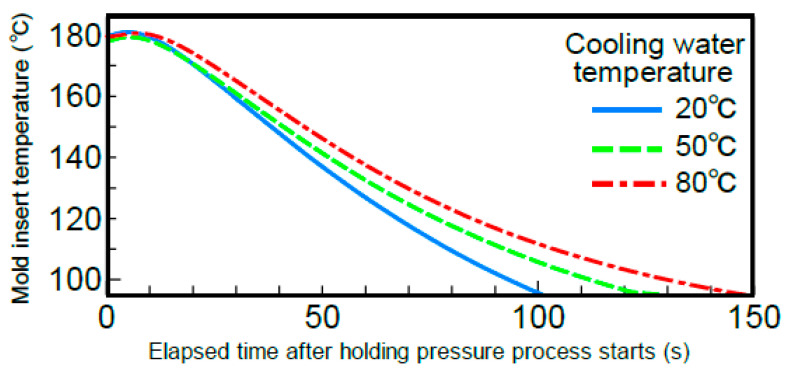
Cavity insert temperature curves for various cooling water temperatures (95→180→95 °C).

**Figure 11:**
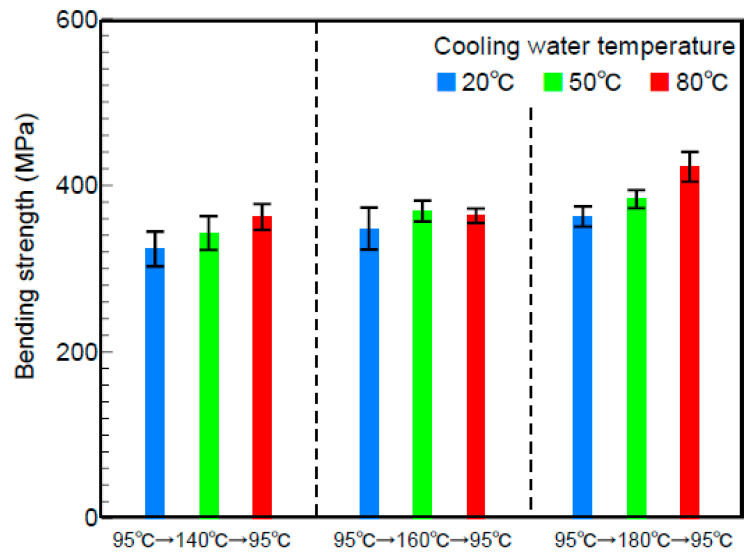
Bending stress for various cooling water temperatures (indenter position ③, melt flow control volume: 15 vol%).

The effect of the cooling speed of the mold on the bending strength in the melt front meeting area was examined. The temperature of the cooling water circulating within the mold was changed to 20, 50, and 80 °C. Figure 10 shows the changes over time at the temperatures measured at C in Figure 2 during heat and cool molding (95 °C→180 °C→95 °C). The figure shows only the results of measurement during the cooling process. The temperature dropped per unit time, namely, the cooling speed of the mold decreased as the cooling water temperature increased. Figure 11 shows the bending strength on line ③ in the melt front meeting area when the cooling speed of the mold was changed by changing the cooling water temperature under different heating conditions of the mold. The bending strength increased as the cooling water temperature increased, namely, as the cooling speed of the mold decreased. The bending strength also increased as the heating temperature of the mold increased. 

The above results indicated that the cooling speed of the mold affected the bending strength.

### 3.4. Observation of Cross Sections of Molded Products

**Figure 12:**
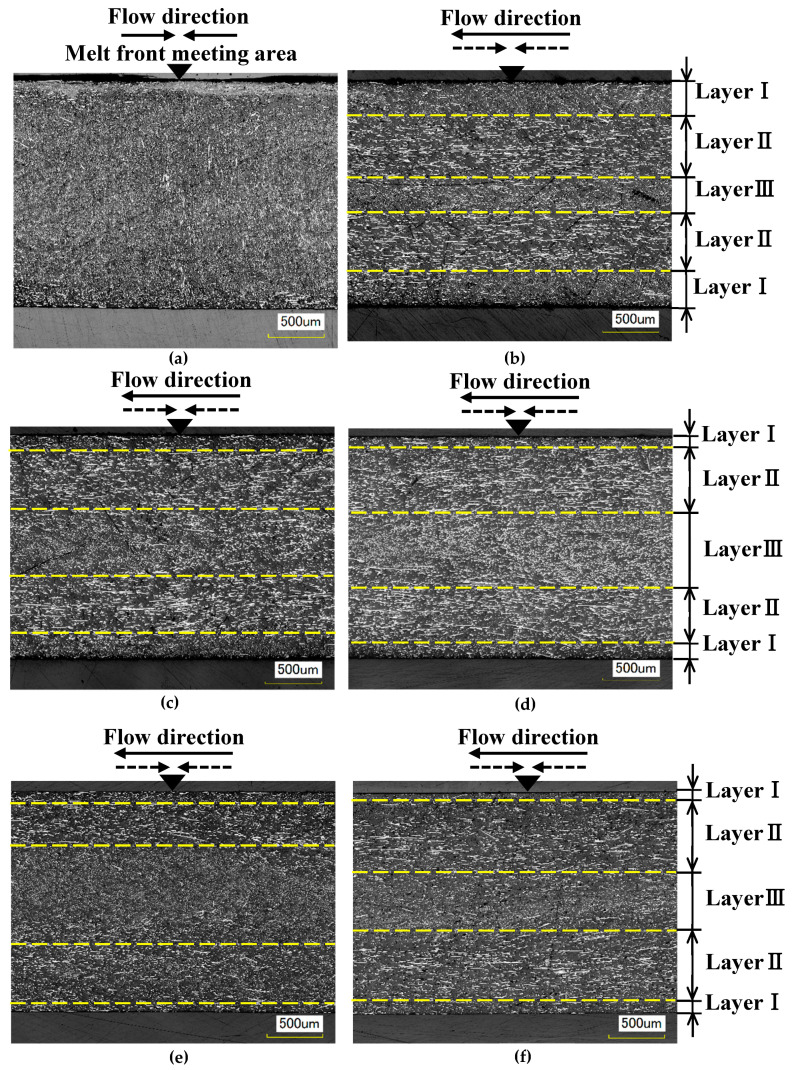
Observation results of fiber orientation along the thickness direction of molded products: (**a**) without flow control (95 °C); (**b**) with flow control (95 °C, melt flow control volume: 15 vol%); (**c**) with flow control (95→140→95 °C, melt flow control volume: 15 vol%); (**d**) With flow control (95→180→95 °C, melt flow control volume: 15 vol%); (**e**) with flow control (95→180→95 °C, melt flow control volume: 5 vol%); (**f**) with flow control (95→180→95 °C, melt flow control volume: 25 vol%).

**Figure 13:**
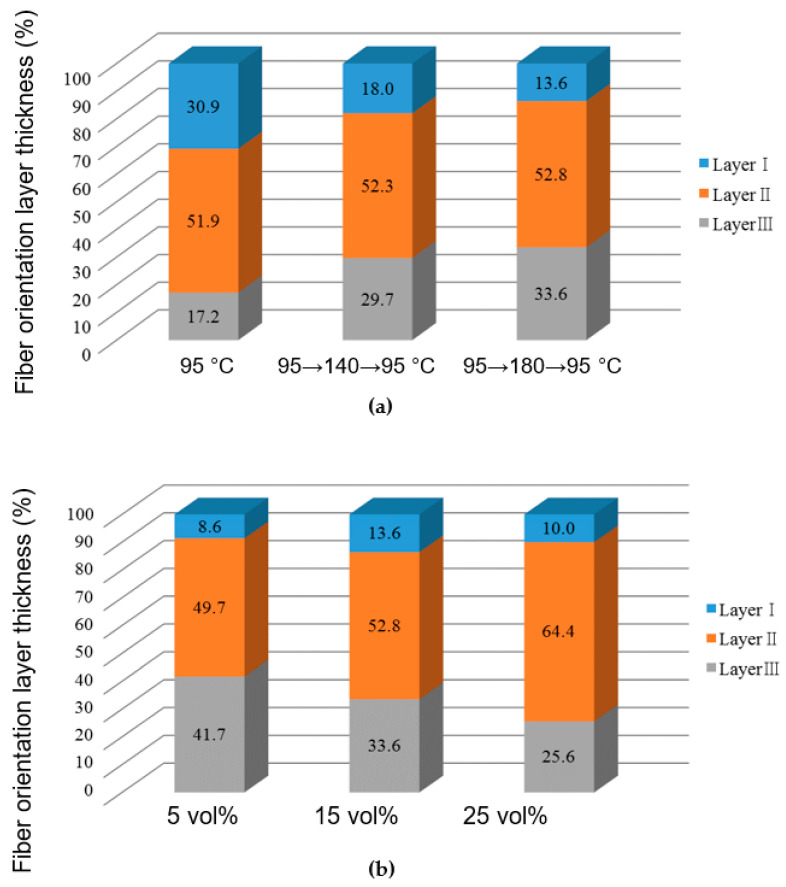
Fiber orientation layer thicknesses as percentages under various molding conditions: (**a**) effect of heating temperature; (**b**) effect of melt flow control volume (95→180→95 °C).

Figure 12 shows the results of observing melt front meeting area ③ on cross section E along the thickness direction of the molded products (Figure 5a). The fibers in and around the melt front meeting area were oriented almost parallel to the meeting area as shown in Figure 12a when the molded product was obtained by two-point gate molding at a constant temperature of 95 °C without melt flow control. On the other hand, three fiber orientation layers (Layers I–III) were formed along the thickness direction of the molded products, as shown in Figure 12b–d, when the molded products were obtained with melt flow control. In Layer I, many fibers in and around the melt front meeting area were oriented almost parallel to the meeting area. Namely, these fibers were oriented perpendicular to the melt flow direction and appeared as dots. In Layer II, many fibers were oriented almost parallel to the melt flow direction. In Layer III, some fibers were oriented parallel to the melt flow direction while others were oriented perpendicular to the melt flow direction and appeared as dots. These three fiber orientation layers were also formed in the molded products shown in Figure 12d–f, which are obtained by heat and cool molding (95 °C→180 °C→95 °C) with different melt flow control volumes. Figure 13 shows the thickness of each layer as a percentage of the total thickness of the mold products, which was calculated on the basis of Figure 12. The thickness of Layer I decreased and the thickness of Layers II and III increased as the heating temperature of the mold increased as shown in Figure 13a. The thickness of Layer I remained virtually unchanged even when the melt flow control volume increased, but the thickness of Layer II increased and that of Layer III decreased as the melt flow control volume increased as shown in Figure 13b.

**Figure 14:**
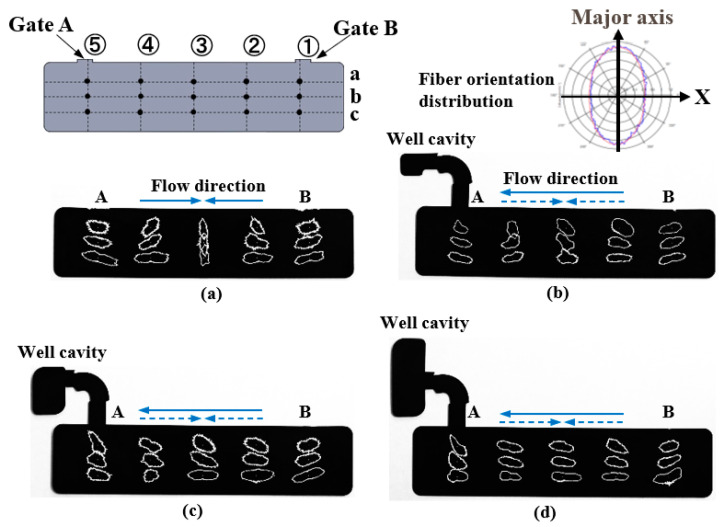
Fiber orientation distribution measured by periodic heating and infrared radiation thermometer method (95→180→95 °C): (**a**) without flow control; (**b**) with flow control (melt flow control volume: 5 vol%); (**c**) with flow control (melt flow control volume: 15 vol%); (**d**) with flow control (melt flow control volume: 25 vol%).

Figure 14 shows the distribution of the fiber orientation at each point on lines ①–④ of the molded products, which was measured by the periodic heating and infrared radiation thermometer method. The molded products shown in this figure were obtained by heat and cool molding (95 °C →180 °C→95 °C). The molded product shown in Figure 14a was obtained by two-point gate molding, whereas the molded products shown in Figure 14b−d were obtained with different melt flow control volumes. The ratio of the length of the long axis to that of the short axis of each ellipse indicated the strength of the fiber orientation. To be more specific, the fiber orientation became more random when the ellipse approached a circle. The angle between the *X*-axis and the long axis indicated the average direction of the fiber orientation. For example, the fiber orientation at point ③−a in Figure 14b–d represented the quantified fiber orientation in the central portion (1.0 mm thickness) of the cross sections along the thickness direction shown in Figure 12d–f, which contained part of Layers II and III. Figure 14 shows that the fiber orientation changed to the melt flow direction in the entire molded product as the melt flow control volume increased. On lines ① and ⑤, which were close to Gates B and A, respectively, fibers were oriented in the direction of the gates in all cross sections. This tendency was stronger at points closer to the gates, such as ①-a and ⑤-a. On line ②, fibers were oriented in the upper left direction at point ②-a. However, the direction of the long axis of the ellipses slightly changed at points ②-b and ②-c regardless of whether or not melt flow control was performed; the fiber orientation was almost parallel to the melt flow direction. On line ③ in the melt front meeting area, the fiber orientation was perpendicular to the melt flow direction in the molded product shown in Figure 14a, which was obtained by two-point gate molding without melt flow control. The fiber orientation gradually changed to the melt flow direction as the melt flow control volume increased. On line ④, the fibers were oriented almost in the upper right or upward direction at point ④-a in the molded product obtained by two-point gate molding without melt flow control and the molded product obtained with 5 vol% of melt flow control. In the molded product obtained with 15 vol% of melt flow control, the fibers rotated anticlockwise and were oriented in the upper left direction because of the melt flow from Gate B to the cavity. The rotation of fibers further proceeded and the fiber orientation changed to the melt flow direction in the molded product obtained with 25 vol% of melt flow control. 

The above results indicated that the thickness of the fiber orientation layers and the direction of the fiber orientation were significantly changed by the heating of the mold and melt flow control.

## 4. Discussion

### 4.1. Appearance of Molded Products

A flat and smooth molded product surface with no exposed fibers and no weld line was generated by heating the mold. Possible reasons for this result are as follows. It has been observed in short-shot molded products that fibers at the melt front are projected from the base material, namely, they are exposed on the surface of the melt, during the fountain flow process. In the normal molding carried out at a constant temperature of 95 °C, the melt cools and solidifies to form a skin layer at the moment the melt forms a fountain flow and comes in contact with the cavity wall. This is because the melt at the melt front is deprived of heat by the mold. As a result, a molded product surface with exposed fibers is formed. Once the surface with exposed fibers is formed, the surface conditions cannot be changed and the weld line cannot be eliminated even by melt flow control. On the other hand, in the heat and cool molding, the solidification of the melt near the cavity wall is slowed because of the high temperature of the cavity wall. The melt does not liquefy at the heating temperature in the experiments because the melting point T*m* of the semi-aromatic polyamide used in this study is 243 °C. However, the skin layer softened by the heat of the mold is pressed onto the cavity wall by the internal melt pressure generated by melt flow control. At this moment, the melt surrounds the exposed fibers before it cools and solidifies. As a result, a flat and smooth molded product surface with no exposed fibers and no weld line is generated.

The formation of an undulating shape with the melt front meeting area as its apex is prevented by melt flow control regardless of whether or not the mold is heated. On the basis of a research report by Mizutani et al. [21], we considered as follows. In and around the melt flow meeting area shown in Figure 12a, fibers oriented along the thickness direction of the molded product had reinforcing effects, which prevented the shrinkage of the molded product in the thickness direction. On the other hand, fibers were oriented parallel to the molded product surface in areas other than the melt flow meeting area, resulting in large shrinkage in the thickness direction. An undulating shape with the melt front meeting area as its apex was formed because the molded product had both the meeting area with small shrinkage and the surrounding area with large shrinkage. An internal melt flow was generated by melt flow control and changed the fiber orientation in the meeting area from the thickness direction of the molded product to the direction parallel to the molded product surface, as shown in Figure 12b–f, regardless of whether or not the mold was heated. As a result, a flat surface was formed because the entire molded product shrank uniformly in the thickness direction.

### 4.2. Cooling Speed of Mold and Bending Strength

The bending strength in melt front meeting area ③ increased as the cooling speed of the mold decreased or the heating temperature of the mold increased. The data reported in Ref. [22] showed that the semi-aromatic polyamide used as a base material was most easily crystallized at 150–180°C because its semi-crystallization time was shortest in this temperature range. The temperature of the melt decreased slowly in this temperature range when the cooling speed of the mold was low or the heating temperature of the mold was high. As a result, the crystallization of the semi-aromatic polyamide was enhanced. It is considered that such enhancement of the crystallization increases the bending strength.

### 4.3. Bending Strength and Fiber Orientation

**Figure 15:**
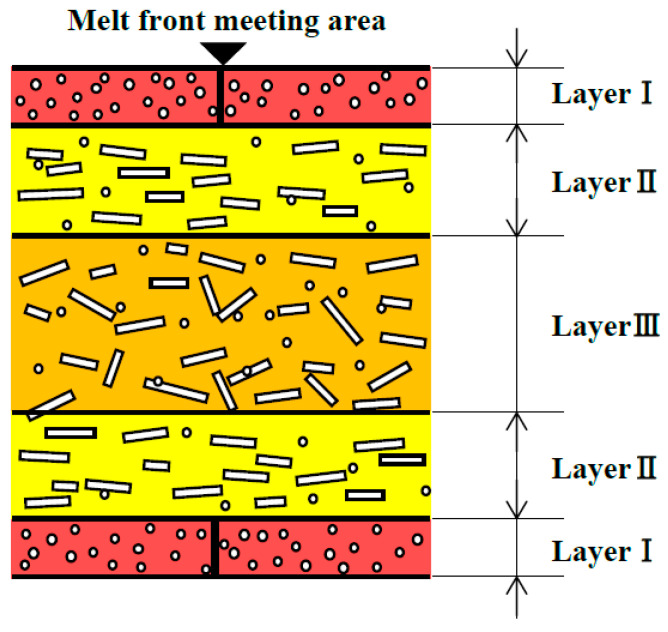
Diagram of fiber orientation layer structure along thickness direction of molded product.

Figure 15 shows a diagram of the fiber orientation in and around the melt front meeting area formed by melt flow control, which was created by the observation of the cross sections of the molded products shown in Figure 12. Layer I was formed when fibers and the surrounding melt moved toward the cavity wall and solidified during the fountain flow process. Layer I contained many fibers that were oriented parallel to the meeting area. When the bending load was applied from the vertical direction to the molded product surface, as in this study, the bending strength was considered to be low in Layer I, in which fibers were oriented parallel to the direction in which the load acted or the longitudinal direction of the indenter. Layers II and III were formed by the internal melt flow generated by melt flow control. A previous report showed that the fibers in a shear flow were oriented parallel to the melt flow direction [23]. Layer II was considered to be formed by a shear flow. For Layer II, we confirmed the distribution of many fibers oriented parallel to the melt flow direction due to shear, or oriented orthogonally to the longitudinal direction of the indenter. Therefore, the bending strength is considered to be highest in Layer II. Layer III was subjected to less shear than Layer II and contained fibers oriented both parallel and perpendicular to the melt flow direction. The bending strength in Layer III was considered to be lower than that in Layer II and higher than that in Layer I.

When melt flow control was performed, the bending strength in melt front meeting area ③ increased as the heating temperature of the mold increased. On the basis of the above discussion, the reasons for this result were considered to be as follows. As the heating temperature of the mold increases, the area of the internal melt flow associated with the shear flow near the cavity wall increases because the solidification of the melt near the cavity wall is slowed. As a result, the thickness of Layer I, having low bending strength, decreases, whereas those of Layers II and III having the highest and second highest bending strengths, respectively, increase. The increased thicknesses of Layers II and III as well as the causes of the enhanced crystallization described in Section 4.2 seemed to be the reasons for the increase in bending strength with increasing heating temperature of the mold. When melt flow control was performed, the bending strength in melt front meeting area ③ increased as the melt flow control volume increased. Possible reasons for this result are as follows. The internal melt flow occurs for a long duration and the region of shear flow extends to the center of the cavity as the melt flow control volume increases. The extension of the region of shear flow results in the decreased thickness of Layer III and the increased thickness of Layer II, where the latter contains many fibers oriented parallel to the melt flow direction and has the highest bending strength. This seems to be the reason for the increase in bending strength with increasing melt flow control volume.

The bending strength in each area of the molded products was examined. The bending strength on line ② did not change and remained high as the melt flow control volume increased. This seemed to be because the fiber orientation on line ② changed negligibly even if melt flow control volume changed. The bending strength in melt front meeting area ③ increased sharply as the melt flow control volume increased, approaching the bending strength on lines ② and ④ when the melt flow control volume was more than 15 vol%. A possible reason is that the fiber orientation in melt front meeting area ③ is significantly shifted to the direction parallel to the melt flow direction as the melt flow control volume increases. The bending strength was high on line ④. The same bending strength was observed on line ④ in the molded product obtained by two-point gate molding and in the molded products obtained with melt flow control volumes of 5 and 15 vol%. The bending strength on line ④ was slightly higher in the molded product obtained with the melt flow control volume of 25 vol%. The fiber orientation on line ④ rotated anticlockwise from the upper right direction to the upper left direction as the melt flow control volume increased. Finally, fibers were oriented along the melt flow direction when melt flow control volume was 25 vol%. There was negligible difference between the bending strength when the fibers were oriented in the upper right direction and that when the fibers were oriented in the upper left direction because the absolute values of the orientation angle were the same. The bending strength increased when the melt flow control volume was 25 vol% because fibers were oriented parallel to the melt flow direction. As discussed above, the bending strength on line ④ seemed to vary according to the changes in fiber orientation. It has been reported that delamination greatly affects the strength of fiber-reinforced composite materials [24]. We confirmed that delamination occurred at the melt front meeting face of a two-point gate molded product, resulting in the breakage of the product. For a one-point gate molded product and a molded product with melt flow control, we confirmed that the part of the molded product to which an indenter was pressed broke parallel to the loading direction and that the fibers on the broken surface were folded or came off, causing the fibers to be exposed. However, we have not yet examined the occurrence of delamination at the interfaces between Layers I and II and between Layers II and III or the effect of delamination on the bending strength of the molded product. As a result, we only discuss the fiber orientation of Layers I–III and their thickness in this paper. We need to discuss in detail the mechanism underlying the breakage of the molded product including the delamination in future work.

## 5. Conclusions

An injection mold capable of melt flow control and induction heating and cooling was used in the injection molding of short carbon fiber reinforced semi-aromatic polyamide. The effects of heating and cooling the mold as well as melt flow control on the appearance, fiber orientation, and bending strength of molded products of the above polyamide were examined. We obtained the following results.

1. By heating the mold, the solidification of the melt in the surface area of the molded product was slowed down and a flat and smooth molded product surface with no exposed fibers or weld line was generated. Moreover, a flat surface without an undulating shape was formed regardless of whether or not the mold was heated because the fibers in and around the melt front meeting area were oriented parallel to the molded product surface by melt flow control.
2. The bending strength in the melt front meeting area increased as the cooling rate of the mold decreased or as the heating temperature of the mold increased. A possible reason is that the crystallization of semi-aromatic polyamide is enhanced.
3. The bending strength in the melt front meeting area increased with the heating temperature of the mold when melt flow control was performed. This may be because the crystallization was enhanced as described in (2). In addition, the thickness of Layer I, which had low bending strength because of fibers oriented parallel to the melt front meeting area, decreased whereas the thicknesses of Layers II and III, which respectively had the highest and second highest bending strengths because of fibers oriented parallel to the melt flow direction, increased.
4. The bending strength in the melt front meeting area increased as the melt flow control volume increased for all heating temperatures of the mold. This appears to be because of the increased thickness of Layer II with the highest bending strength. The bending strength varied in the entire area of the molded products in accordance with the changes in fiber orientation.

As discussed above, the effects of both heating the mold and melt flow control on the appearance and bending strength of the molded products were systematically evaluated using the injection mold capable of melt flow control and induction heating and cooling. This injection mold can be applied not only to glass and carbon fibers but also to filler-filled materials such as cellulose nanofibers. Further studies will be carried out using this injection mold.

## Figures and Tables

**Table 1:** Molding conditions.

Cylinder Temperature	(°C)	280-280-275-270-80 ^*^)
Mold temperature	(°C)	(1)95
		(2)95→140/160/180→95
Injection rate	(cm^3^/s)	23.9
Holding pressure	(MPa)	80
Holding pressure period	(s)	15
Melt flow control volume	(vol%)	5/15/25

^*)^ Nozzle-Metering-Compression-Feed zone-Under hopper.

## Data Availability

Not applicable.
